# Towards a Comprehensive and Robust Micromanipulation System with Force-Sensing and VR Capabilities

**DOI:** 10.3390/mi12070784

**Published:** 2021-06-30

**Authors:** Georges Adam, Subramanian Chidambaram, Sai Swarup Reddy, Karthik Ramani, David J. Cappelleri

**Affiliations:** School of Mechanical Engineering, Purdue University, West Lafayette, IN 47907, USA; schidamb@purdue.edu (S.C.); reddy37@purdue.edu (S.S.R.); ramani@purdue.edu (K.R.)

**Keywords:** micromanipulation, microrobotics, force sensing, virtual reality

## Abstract

In this modern world, with the increase of complexity of many technologies, especially in the micro and nanoscale, the field of robotic manipulation has tremendously grown. Microrobots and other complex microscale systems are often to laborious to fabricate using standard microfabrication techniques, therefore there is a trend towards fabricating them in parts then assembling them together, mainly using micromanipulation tools. Here, a comprehensive and robust micromanipulation platform is presented, in which four micromanipulators can be used simultaneously to perform complex tasks, providing the user with an intuitive environment. The system utilizes a vision-based force sensor to aid with manipulation tasks and it provides a safe environment for biomanipulation. Lastly, virtual reality (VR) was incorporated into the system, allowing the user to control the probes from a more intuitive standpoint and providing an immersive platform for the future of micromanipulation.

## 1. Introduction

Micromanipulation has gained a lot interest in recent years, especially due to an increasing demand for systems capable of reliable and accurate micropositioning and sensing [1,2]. Some of the applications for such systems include biomanipulation [3,4], cell/tissue characterization [5,6], micro-assembly [7], among others. For many biomedical applications there is a need for robust and accurate systems, since the objects being manipulated (usually cells or tissues) are extremely fragile and highly susceptible to damaging forces during manipulation. With the development of the micromanipulation field, it is trending towards more complex systems with multiple functionalities, such as sensing and grasping. This can be achieved through the development of specialized end-effectors and the increase of controllable elements in the system, utilizing different end-effectors in conjunction to achieve more complex tasks.

The micromanipulation field can be divided into two main categories: tethered and untethered systems. Untethered, sometimes referred to as field-driven micromanipulation, replaces mechanical manipulator components with components that generate a field (acoustic [8,9,10], magnetic [11,12,13], laser/light [14,15], etc., which in turn cause manipulation directly or actuate an untethered end-effector, usually in the form of a microrobot. For tethered systems, the manipulation end-effector is attached mechanically to the actuation element, typically DC or stepper motors. In most cases, untethered systems have more degrees-of-freedom (DOFs) than tethered systems, which are usually constrained in their rotation, however they present lower positional resolution. Furthermore, the range of applications is slightly reduced in untethered systems since sometimes the actuation field can interfere with the material being manipulated, causing undesired results. Here, a high resolution, 3 DOF micromanipulators are used in conjunction with several end-effectors, tackling several different possible applications.

In order to increase the complexity of tasks and dexterous capabilities of micromanipulation systems, multiple micromanipulator probes (or fingers) and end-effectors are used together [16,17,18,19,20]. Additionally, force sensing capabilities allow the system to be used for many delicate applications that have strict allowable safe force thresholds during manipulation, such as the case for biomedical applications. With an embedded force sensing unit, the system is capable of ensuring the threshold force is never surpassed and in the case of a biological medium, the object being manipulated retains its properties and viability. Multiple force sensing methods have been shown for micromanipulation or microrobotic applications, such as piezoelectric/piezoresistive [21,22,23,24], atomic force microscope (AFM) [25,26], vision-based [11,27], and capacitive [28,29]. From these methods, a vision-based force sensor modality is selected for use here since it is able to overcome many of the drawbacks other sensors, such as high costs and difficult integration with micromanipulation systems (AFM sensors), complicated circuitry (capacitive sensors), and temperature sensitivity (piezoresistive sensors). By using a compliant structure with known stiffness along with a vision-system for tracking, micro-force sensing can be achieved by computing the deflection of the calibrated structure. This method does not require any electrical components, has a small footprint, and can be incorporated easily into different micromanipulation test-beds.

In an effort to make the overall system and micromanipulation more intuitive for the user, the integration of haptic devices or novel control input mechanisms has been used. With the increased complexity and capabilities of virtual reality (VR) devices and systems, they have become a potential solution for improving the capabilities of the micromanipulation systems, while giving more control and a greater manipulation “feel" to the user. Some of the capabilities that it improves are the potential for higher repeatability and the creation of a high fidelity simulation environment for training purposes.

In this paper, a micromanipulation system with integrated 3D vision-based micro-force sensing probes is presented for the first time. In this system, multiple probes can be actuated individually or simultaneously in a coordinated fashion to achieve and simplify more complex manipulation tasks while providing force feedback to the user. A graphical user interface (GUI) was developed as a robust and comprehensive platform to intuitively control the entire system and its many capabilities. Furthermore, a VR system has been implemented to provide intuitive manipulation, as well as to unlock new future capabilities for the system.

## 2. Materials and Methods

### 2.1. Micromanipulation System Overview

**Figure 1:**
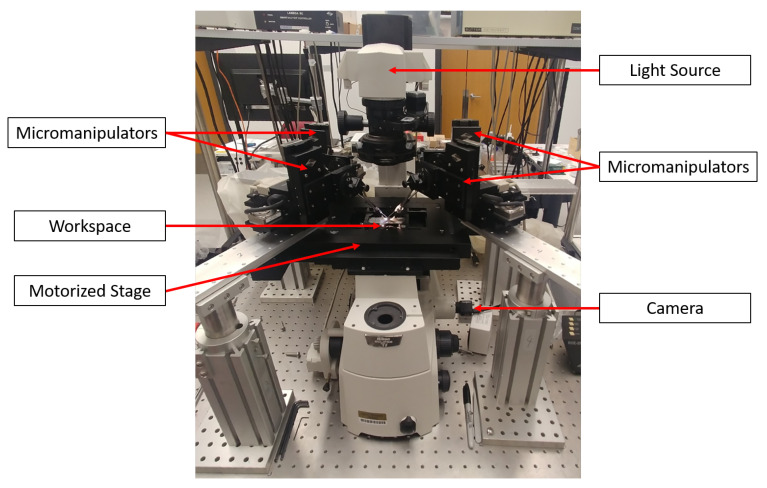
The vision-based micro-force sensing micromanipulation system (μVBFS-MS). Up to four micromanipulators can be mounted around the inverted optical microscope system with a integrated motorized XY stage. Custom end-effectors for the micromanipulators allow for 3D vision-based micro-force sensing in conjunction with tracking algorithms operating on real-time images captured with the camera system.

**Figure 2:**
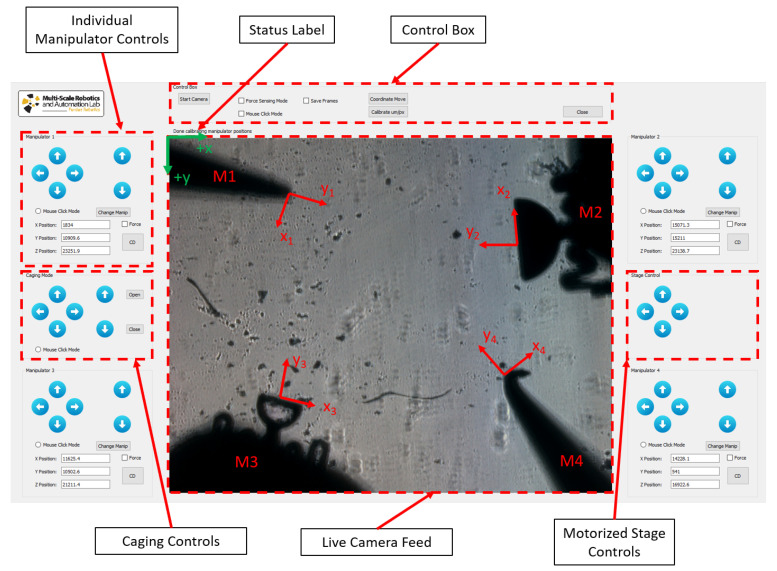
Workspace view and custom graphical user interface (GUI) for manipulator and stage control. In this case, a workspace configuration with two blunt probes (M1 and M4) and two force sensing probes (M2 and M3) is shown. The local coordinate frames for each manipulator (x${}_{i}$, y${}_{i}$) are shown on their respective end-effectors. The global camera frame coordinate system is shown in the top-left corner (green).

The vision-based micro-force sensing micromanipulation system (μVBFS-MS) is built upon a inverted optical microscope (Nikon Eclipse Ti, Nikon Instruments, Tokyo, Japan) with an integrated motorized XY stage (Nikon TI-S-EJOY, Nikon Instruments), and custom mounts for the micromanipulators and custom end-effectors. Figure 1 shows the general configuration of the system with four micromanipulators (MP-285, Sutter Instruments) in the workspace. Each of the manipulators has 3 degree-of-freedom with a travel distance of 1” along each orthogonal axis. The resolution of movement along each axis is 0.04 μm/step of the embedded stepper motor. A camera (1.3 MP CMOS, PointGrey e2v EV76C560) is located underneath the workspace along with an overhead light source and thus only captures the shadow of the objects in the workspace (as seen in Figure 2). This configuration prevents the use of color tracking methods for with vision-based micro-force sensors that were utilized previously [11,27]. Therefore, new tracking methods have been employed in its place, as described in the later sections.

Depending on the desired application, the system can be setup in multiple different configurations, making it a flexible and versatile system with a large breath of applications and possible customization. The inverted microscope test-bed can hold up to 4 micromanipulators that can be used simultaneously. The end-effectors of the probes can be easily and rapidly swapped out depending on the desired application. For the force sensing end-effector, the stiffness of the compliant structure can be tailored based on the soft polymer’s mixing ratio, thus effectively controlling the range and resolution of the sensor. For other applications that do not require force sensing, the probe tips can be replaced by blunt tips, which allow for precise point pushing manipulation. These tips can also be utilized together. As an example, blunt tips can secure an object of interest in place while a force sensing probe pushes on it with specific forces in order to compute the stiffness of the object in question.

Currently, all manipulation happens on a glass slide working surface, however its size and coatings can be adjusted. The only requirement for the workspace surface is that it is transparent so the camera system is able to record the parts on top of the surface. One of the main problems with manipulation at small scales is the often unpredictable effect of friction and other surface forces, which are elevated due to scaling laws. In the past [27], it has been shown that using a hydrophobic surface can lead to lower surface forces and, in turn, more reliable micromanipulation. Lastly, the workspace is mounted on top of a stage, which can change its position with ease. This is specially useful when performing caging applications, in which the manipulator probes completely surround the part and lift it off the surface, This way, the stage can move to the desired end location of the part, and the manipulators simply drop it in place. In this case, force sensing can be used to make sure enough force is being applied in order to lift and hold the object.

### 2.2. Graphical User Interface (GUI) Overview

In order to control all the manipulators and provide a robust toolbox that explores multiple capabilities of the system, a custom Graphical User Interface (GUI) was created using Qt and C++. As shown in Figure 2, the GUI provides extensive information to the user and is able to operate in multiple modes. The status label between the camera feed and the control box provides the user with current status of manipulators, as well as important information, such as forces in real-time. There are four major panels for single manipulator control (one for each manipulator). In each of them, the current X, Y, and Z coordinate positions of the manipulator are shown in microns based on each manipulator’s coordinate frame, as well as buttons for different modes of motion. After editing the text box for the current manipulator coordinate positions, the user can select the *Coordinate Move* button in the *Control Box* panel and the manipulators will move to the newly selected coordinates. Moreover, using *Mouse Click Mode* radio button, the user is able to simply select a manipulator, click on a desired position on the workspace, and the manipulator automatically moves to that location. Lastly, the arrow buttons move the respective manipulator by a fixed increment distance (set by the user) in the arrow direction. In each panel, the four buttons on the left perform the movement on the XY plane and with respect to the camera coordinate frame. The up and down arrows on the right side of the panel perform movements in the z-direction, moving the probes towards or away from the workspace. These take into account the fact that each manipulator is angled and movement in these directions is executed with respect to the camera’s coordinate frame.

For accurate actuation and control of the probes in many of the motion modes mentioned above, there are two calibration values that play a crucial role related to the transformation matrix between the manipulators coordinate frame and the camera coordinate frame. These are the manipulator angle, $\theta_{i}$, which is measured from the horizontal plane, and the spacial resolution of the vision system or μm/pixel ratio. The calibration of these values can be done manually based on an image of the workspace and a calibration slide with known distances on it or using the automated calibration mode option. The latter consists of the system moving each manipulator by a set amount with the user clicking on the initial and final position of each manipulator. This allows the system to accurately compute the manipulator angle and spacial resolution and store these values for future use. Therefore, calibration is only necessary when a manipulator is replaced or some other aspect of the system is modified.

Additionally, the GUI also provides the user with caging and stage controls, as seen on either side of the live camera feed in Figure 2. In this paper, the term caging is used to refer to a squeezing caging grasp, as defined by Rodriguez et al. [30]. The stage controls simply move the motorized stage in the x and y directions. For the caging control, the arrows show the direction that the entire cage assembly will move. In this panel, there are also two extra buttons (“Open” and “Close”), which serve as a fine tuning mechanism for the user when performing the initial cage around the part to be moved. Lastly, the user can also use the *Mouse Click Mode* to control the entire cage assembly, moving it with ease and precision around the workspace.

A few other advanced modes are available to the user in the GUI. These include the *Force Sensing Mode* and *Contact Detection Mode*. For the force sensing mode, the user tells the system which manipulators are equipped with a vision-based micro-force sensing probe by checking the respective box. This way, once the force sensing mode is activated, the forces applied by these manipulators are computed for every video frame. For instance, in Figure 2, the workspace is equipped with two vision-based micro-force sensing probes (M2 and M3). For the contact detection mode (CD button), the selected manipulator will move forward until a force is detected, signaling it is in contact with a part in the workspace. This is especially useful when using the bottom-view camera, since it is hard to detect contact with parts visually. The force sensing mechanism, as well as its working principle, will be discussed in the next section.

### 2.3. The Vision-Based Micro-Force Sensing Probe (μVBFS-P)

The micro-scale vision-based micro-force sensor (μVBFS) has a simple working mechanism and does not require electricity or complicated circuitry. Additionally, since it has a small footprint, it can be easily be mounted to a probe and adapted to various types standard test-beds. The μVBFS works by measuring the deflections of a compliant structure of known stiffness using a camera system and then computing the force according to Hooke’s law. In order to achieve multi-dimensional micro-force sensing, the vision system must be able to track deflections in multiple dimensions and the compliant structure must be calibrated in these directions as well. In this case, the 3D vision-based micro-force sensing probe (μVBFS-P) consists of a rigid body that is attached to the micromanipulation system, a rigid tip for manipulation, and a compliant spring-like structure connecting the two. Figure 2 shows the workspace camera view of two force sensing probes (M2 and M3). The compliant structure is made out of polydimethylsiloxane (PDMS), a soft elastometer with tailorable stiffness. During its fabrication procedure, the PDMS gets its consistency from mixing the PDMS monomer with a curing agent. By changing the weight ratio of these two components, one can effectively control the stiffness of the resulting structure. Here, the PDMS structures were fabricated using a 10:1 ratio.

The rigid parts of the sensor (body and end-effector) are made out of silicon. The entire sensor is fabricated using standard microfabrication techniques, such as photolithography and etching. The compliant structures are created by etching the spring-like shape onto a silicon wafer, creating a trench in which the PDMS is poured in and cured. Then, several photolithography steps followed by etching are performed to etch the shape of the sensor out of the wafer, concluding with a backside etch to release the sensors altogether. All of the etching is done using a deep reactive-ion etching (DRIE) technique, which results in accurate etching with high aspect ratio for the sidewalls. In order to attach the sensor to a micromanipulator probe, a 3D printed attachment structure has been developed. This attachment piece is screwed into the micromanipulator probe on one side and the μVBFS is manually attached using superglue to the other side. Small grooves on the underside of the attachment piece help to keep it aligned with the manipulator itself. The fabrication procedure is described in detail in [27], and a summarized schematic is presented in the Supplemental Materials as Figure S1.

In order to calibrate the 3D stiffness of the sensor, it was fixed on a glass slide and a micromanipulator was used to push a MEMS capacitive force sensor (FT-100, FemtoTools) into the end-effector. The micromanipulator is able to measure the distance travelled (and thus deflection of the compliant structure), while the MEMS force sensor continuously measures force information. A calibration plot of force versus deflection is then created and the slope measured, which in turn is the directional stiffness of the compliant structure. Note: for the small deflection regime that the sensor is operating in, a linear model of the stiffness is sufficient. By rotating the sensor, the same procedure is repeated to compute the stiffness in all 3 directions in the sensor’s frame of reference (x${}_{s}$, y${}_{s}$, and z${}_{s}$). The fabrication procedure and calibration of the μVBFS is the same as in a previous work in which the sensor was introduced [27]. In the future, a calibrated force sensing probe can be used to calibrate other probes. Since the system provides a high level of movement accuracy, one μVBFS-P can accurately perform the function of the MEMS force sensor in the original calibration procedure. Table 1 shows the properties of two sensors used. This shows the great range of forces that these sensors are able to apply, which can be specifically tailored to a target application during the fabrication of the compliant PDMS structure.

As previously mentioned, color tracking algorithms cannot be used since the bottom-view camera and top-down light source only produces a silhouette image of the workspace. Therefore, we investigated and analyzed different tracking methods for the current application, namely the MOSSE [31], CSRT [32], and Median Flow [33] methods. Between these, CSRT was the most accurate method, however also the slowest. MOSSE was the fastest method, but was not reliable enough to provide robust tracking. Lastly, the Median Flow method was able to provide a good balance between speed and robustness, thus it was used for the experiments described below. In order to track multiple probes simultaneously, the camera frame is divided into smaller cropped images of regions of interest (ROIs) around each force sensing manipulator. Each of these ROIs is rotated by the manipulator angle (to be perpendicular to the camera frame) and the tracking algorithm is applied to the spring-like structure of the probe. Using this technique, from measuring the changes in the height and width of the structure, its deflection can be computed. This allows for a straight-forward approach to obtain the sensed forces since the measured deflection (in μm) can simply be multiplied by the calibrated stiffness (in N/m) of the probe in the respective direction (x${}_{s}$, y${}_{s}$, or z${}_{s}$).

In this system, the algorithm tracks the deflections of the sensors ($\delta_{xs}$, $\delta_{ys}$, $\delta_{zs}$) by rotating the 2D camera view into the desired manipulator’s local coordinate frame (x${}_{i}$, y${}_{i}$) and it converts them into the sensor’s 3D coordinate frame (x${}_{s}$, y${}_{s}$, z${}_{s}$) to compute the forces. At first, the algorithm obtains the deflections within the local manipulator coordinate frame ($\delta_{xi}$, $\delta_{yi}$). These deflection values need to be converted into deflection values in the sensor coordinate frame ($\delta_{xs}$, $\delta_{ys}$, $\delta_{zs}$) so they can be multiplied by the respective sensor axis stiffness and the force calculated.

**Figure 3:**
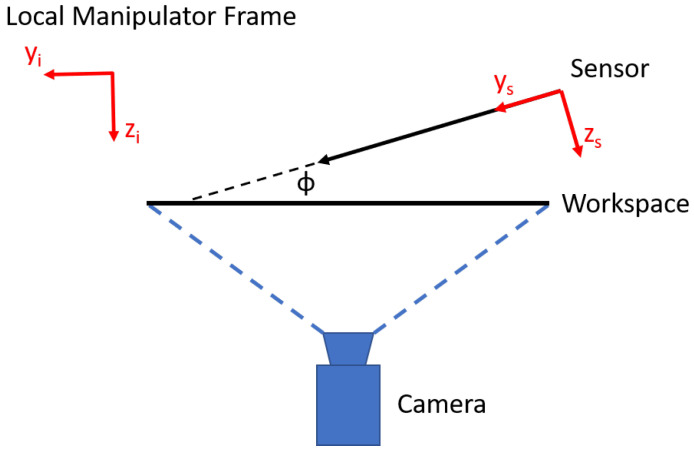
Schematic showing a side view of the system with the coordinate frames used to compute the forces.

Let ϕ be the out-of-plane angle between the sensor and the workspace (Figure 3). Note that here, the deflection in the x-direction of the local manipulator frame ($\delta_{xi}$) corresponds to the x-direction deflection of the sensor in the sensor coordinate frame ($\delta_{xs}$). Therefore, only the y-deflection ($\delta_{yi}$) in the manipulator frame needs to be decomposed into the sensor’s frame of reference. Writing down the displacement equations in the manipulator coordinate frame, we get:

(1) $$\delta_{xi}=\delta_{xs}$$

(2) $$\delta_{yi}=\delta_{ys}\cdot cos\left(\phi \right)-\delta_{zs}\cdot sin\left(\phi \right)$$

The measured deflection in the manipulator’s local frame ($\delta_{yi}$) represents a projection of the deflection in the sensor’s coordinate frame in along the y${}_{s}$ and z${}_{s}$ axes. To solve Equation (2) for $\delta_{ys}$ and $\delta_{zs}$, it is assumed that the out-of-plane angle (ϕ) is small enough that all of the deflection happens along the sensors y-axis (y${}_{s}$). This is a valid assumption since for accurate caging micromanipulation, the angle ϕ must be kept small, as discussed later in the manuscript. If the angle is too steep, then most of the force would be applied along the sensor’s z-direction ( z${}_{s}$), causing the part to slip and fall out of the caging assembly. Using this assumption, $\delta_{zs}$ is set equal to zero and Equation (2) can be easily solved for $\delta_{ys}$ and $F_{ys}$ subsequently determined, as shown in Equations (3) and (4).

(3) $$\delta_{ys}=\frac{\delta_{yi}}{cos(\phi )}$$

(4) $$F_{ys}=k_{ys}\cdot \delta_{ys}$$

### 2.4. Virtual Reality (VR) System

**Figure 4:**
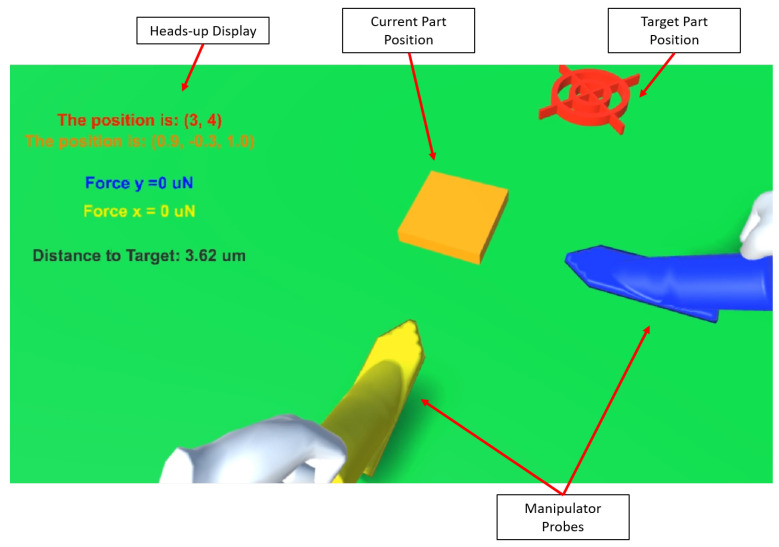
The VR scene as seen from the user’s point of view. The heads-up display shows important information to the user and it always remains on the top left side of the view. The part’s current and target positions are represented by the orange square and the red cross hairs, respectively.

A VR application was developed to enable a more intuitive and spatial method for the manipulation of the actuated micromanipulation probes. As shown in Figure 4, the VR application provides a scaled representation of the workspace, with two force-sensing probes. This application allows the user to spatially interact with the virtual workspace; then these changes are reflected in the real workspace through the control of the actuated probes. The VR application also provides the necessary feedback required by the user during the manipulation process including probe positions, force sensor readings, distance to target, and real-time video feedback.

This system was deployed using an Oculus Quest 2 (Oculus, Facebook Technologies, LLC, Irvine, CA, USA) headset in conjunction with a VR-Compatible PC connected via an Oculus Link cable. The Oculus Quest 2 is powered by a desktop computer with an Intel Core i7-9700 processor and a NVIDIA GeForce RTX 2060 GPU. The system was developed with Unity3D 2018.3.14f1. To allow interaction with the virtual replicas of the actuated probes, we used virtual hand representations that were bound to the Oculus Controllers via the Unity Oculus SDK (Oculus, Facebook Technologies, LLC).

In the VR application, the user is able to manipulate the position of the actuator probes by moving the corresponding virtual models using the Oculus handheld controllers. The user is able to perform the manipulation in the VR environment and store a series of checkpoints that represent the full motion path of each actuated probe involved in the manipulation. The user directly manipulates the virtual probe models and their full path is recorded. This recording can be played back to the user in the VR environment, or sent to the physical micromanipulation system. This process entails sending all of the path coordinates of the manipulation to the physical system, which in turn maps the virtual motions and transforms them into instructions that represent the same motion. With the use of the force sensing probes, the user is able to select a maximum threshold force to keep the manipulation process safe. If this force is exceeded in the real system at any time, the manipulation process is halted.

## 3. Results

As previously described, a robust and multifunctional micromanipulation system with 3D micro-force sensing capabilities has been developed. In order to showcase a few of its current capabilities and possible future work extensions, several experiments were conducted, as described below. Automatic contact detection is demonstrated first. Then simple and complex caging applications are presented to show the versatility of the system. Finally, the current VR capabilities are provided as a proof-of-concept for future generations of the system.

### 3.1. Contact Detection

One challenge of using a bottom-view camera with parts and probe tips possible located at different focal planes is that it can be difficult to tell if a probe is in contact with an object to be manipulated. The object and probe tip may visually appear to be in contact from the camera view when in reality on their shadows are in contact and/or the probe tip and part are actually in different vertical planes. Since the probes in this system have force sensing capabilities, an automatic contact detection mode was developed to aid users with fine tuning the approach to an object. In this mode, the selected manipulator moves forward incrementally until forces are sensed, thus signifying the successful contact with the part.

**Figure 5:**
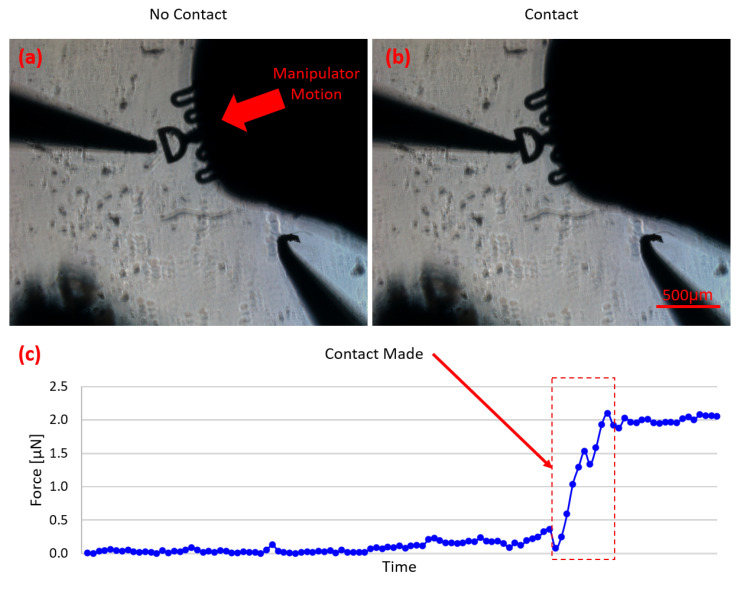
Summary of automated contact detection algorithm. (**a**) The force readings are close to zero, so the manipulator keeps moving forward in small increments. (**b**) Contact has been detected so the manipulator halts its motion and ends the contact detection routine. (**c**) Plot showing the measured forces during this procedure. The point of contact can be clearly identified from examination of the sensed forces.

As shown in Figure 5, the selected manipulator keeps moving in small increments until contact has been made. By looking at the measured force profile over time (Figure 5c), the point of contact is clearly shown. Depending on the size of the object being manipulated, the minimum force requirement to consider successful contact can be updated, since larger parts present more friction force and allow for greater force application before motion is initiated. This specific detection mode is incredibly useful for many micromanipulation applications, especially in when establishing a squeezing caging grasp case since all probes must be in contact with the part in order to initiate a successful 3D caging transport primitive.

This feature can not only be used as a contact detection tool but it can also be turned into a precise force applicator since the probe will continue to move forward until the desired force threshold is reached. This way, the object of interest can be placed in between a stationary probe, acting as a stopper, and the μVBFS-P, allowing for arbitrary micro-forces to be applied. This opens up multiple applications such as mechanical characterization of soft tissues and biological cells, and mechanobiology studies, among others.

### 3.2. Caging Accuracy

Using all of the motion features and actuation modes the GUI offers, the user is able to easily and intuitively move the probes around the workspace. Since surface forces are predominant and very hard to quantify in the micro/nano scale, pushing manipulation not always the most robust approach. One way to solve this problem is by using squeeze caging manipulation transport primitive. Here, the manipulators surround the desired part and apply a sufficient force to it from all sides in order the grasp it. The cage is then actuated to lift the part from the surface of the workspace by simultaneously moving all probes in the vertical (+z) direction. By then moving simultaneously in the XY plane, the manipulators can bring the cage assembly above the goal location with ease, at which point the part can be lowered to its goal location.

In order to test the accuracy of this manipulation method, a part was moved following the shape of a square with 200 μm sides. Then, the actual position of the centroid of the part was compared to its nominal location based on the programmed path. This experiment was repeated for both polygonal and circular objects. The same was done for a simple push manipulation protocol using a blunt tip probe. In this case, the part was pushed in a straight line and the deviations from the nominal path were also measured. These experiments were repeated multiple times for each method. Table 2 shows the results from these experiments comparing the accuracy of each manipulation method. The offsets reported here are the difference between the nominal position of the centroid of the part and its actual position after a movement. As expected, caging has been proven to be a much more reliable transport method for both types of parts since it is able to negate most of the micro-scale surface forces. It is clear that these forces play a large role in push manipulation in the micro-scale, being one of the biggest contributing factors for the high standard deviation in the results. On the other hand, using the cage method, the results show that the manipulation process is much more controllable and offsets are small, even for large distance manipulations. Furthermore, this also confirms that the manipulator calibration is adequate, since the probes move as expected, closely following the projected path for the caging method. Note: While both part shapes tested 2D, we would expect similar accuracy for 3D spherical shapes when grasping them at their midpoint. The compliance of the force sensor will accommodate for some vertical misalignment off the center of the sphere. If the spherical object is too heavy, it may cause the force sensor to buckle. However, an appropriately stiff force sensor can be selected based on the application requirements.

**Figure 6:**
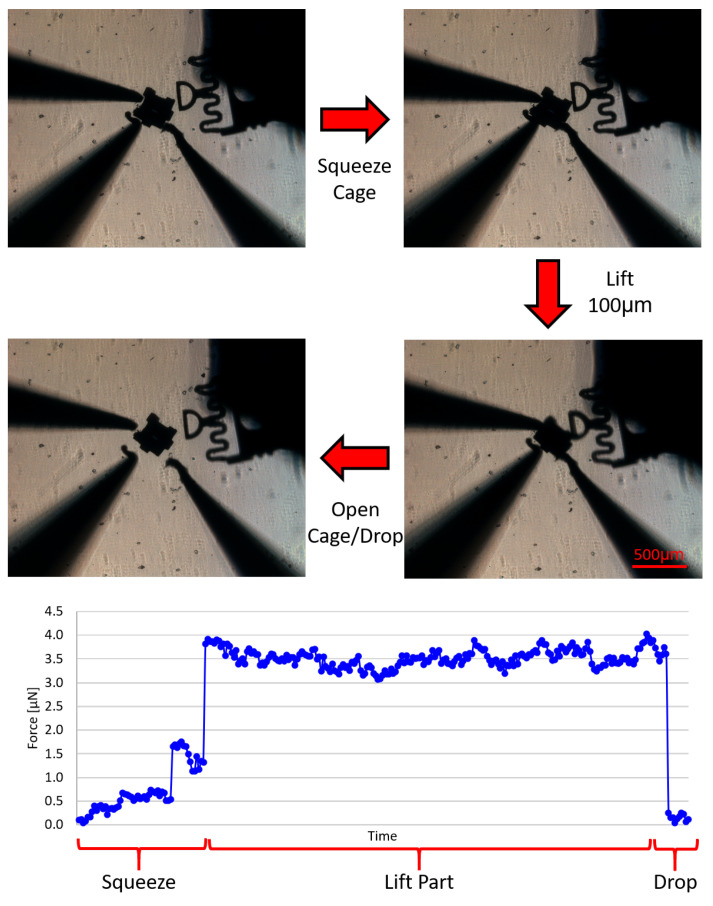
Schematic of the caging protocol. Here, the part is squeezed until the threshold force is surpassed, then lifted off the workspace surface, and released once the manipulation is completed.

As a method to keep the results consistent between experiments, a squeeze caging protocol has been devised to perform the lifting and manipulation. First, the manipulators are placed around the part to completely surround it, but without touching it. Then, the “Close” button in the cage controls panel of the GUI is used to establish the squeeze cage. Once the force is above a certain threshold (for this part it is approximately 2.5 μN), the user can be sure that the part has been successfully caged. This force is different based on the part being manipulated, and it ensures the force is strong enough to lift the part without causing it any damage. Then, all manipulators are moved up (in the z-direction) simultaneously, moving the part off the surface of the workspace. Once the desired position is reached, the “Open” button is pressed, opening the cage and releasing the part. Figure 6 shows a summary of the caging process with its force information. As seen in the force plot, during the squeeze portion of the process, the forces incrementally increase based on the cage closing, then they remain constant after the cage has been secured, and once it is released, no more forces are applied.

### 3.3. Caging Manipulation/Assembly

**Figure 7:**
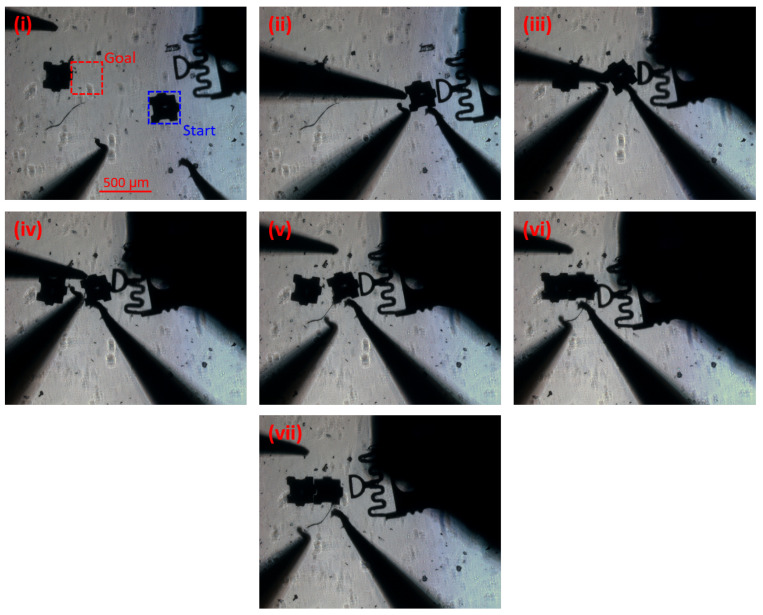
Summary of the 2D assembly process using the squeeze caging primitive. (**i**) Initial setup with starting and goal locations shown; (**ii**) Squeeze cage formed. Note: the position of the part changes slightly between (**i**) and (**ii**) because when the squeeze cage is being formed, the initial spacing between the part and the probes is slightly different, causing some part manipulation prior to the formation of the cage. (**iii**) and (**iv**) show the manipulation; (**v**) Retraction of manipulators 1 and 3 (M1 and M3); (**vi**) Two-manipulator positioning and angle fine tuning; (**vii**) Probe retraction after the assembly is complete.

In order to test the caging protocol in a more realistic setting, two assembly experiments were performed. First, a simple 2D caging assembly was performed (similar to a pick and place application) as a proof of concept of the capabilities of the system. In this case, the user can take advantage of any of the motion tools the GUI provides to get the probes close to the part to be moved. Figure 7 shows a summary of this process, in which the blue square represents the initial position and the red square the final position. Using the devised caging protocol, the part is surrounded and squeezed (ii), then moved closer to the goal location (iii and iv). Here, all manipulator probes are moved simultaneously, using the cage control panel in the GUI, to maintain the squeeze cage. Manipulators 1 and 3, on the left, are retracted for final push (v), allowing manipulators 2 and 4 to perform the fine tuning of the orientation of the part and final push (vi), completing the assembly. The fine tuning of the orientation of the part is a manual process in which the user utilizes a probe to push the part off its center of mass, resulting in its rotation. Furthermore, this process can be performed autonomously using two probes: one to fix a corner of the part and the other to perform the push, effectively creating a pivot point around the fixed corner. This is similar to the rotational motion primitive shown in Cappelleri et al. [34]. After this, the manipulators back out and the assembly is complete (vii). In this assembly, the part is moved approximately 1.5 mm from initial to final position.

**Figure 8:**
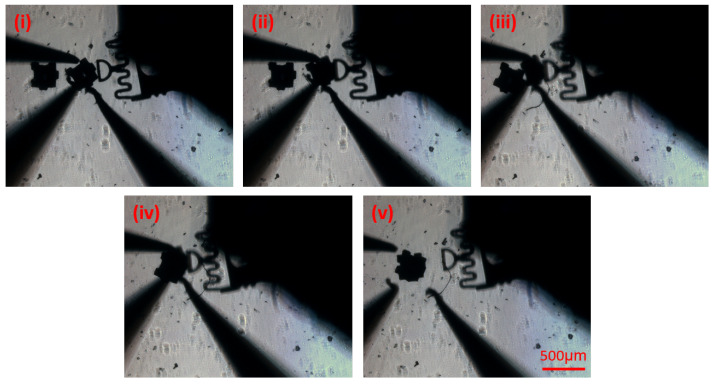
Summary of the 3D assembly (stacking) using the force-assisted cage primitive. Here, (**i**) represents the initial position, (**ii**), squeeze cage, (**iii**) and (**iv**) lift and move part, (**v**) drop and open cage.

To further showcase some more complex capabilities of the system, a 3D assembly, or stacking, was performed. Here, the same caging protocol was utilized and the part was lifted by 150 μm off the surface, allowing it to be placed on top of the other part in the workspace. Figure 8 shows a summary of the stacking process. In this case, (i) shows the initial position, followed by squeeze caging (ii), and lifting of the part (iii). Then, the part is moved above the target part (iv), and finally dropped using the “Open” push button (v). At the end of the process, the orientation of the part is slightly adjusted manually, using the same motion primitives and process as the orientation fine tuning shown at the end of the 2D assembly process.

### 3.4. VR Experiments

In order to help immerse the user in the micromanipulation and make the process more intuitive, VR capabilities were added to the system. This feature allows the user to perform the manipulation in a safe, virtual environment, and then send the command to the real-life system to reproduce the VR motion. In cases in which sensitive parts are in play, the user can set up a maximum force value that the system may not exceed as to not damage the objects. This way, once the instructions are sent to the system, micromanipulation will follow as long as the measured forces are below the set threshold. In the case the forces get too high, the process is automatically halted and the user receives a status message. Figure S2 in the supplemental materials shows a schematic of the VR system functionalities.

To demonstrate this capability, a simple micromanipulation task was performed using two force sensing probes. In the first instance, the forces remained below the set threshold and manipulation was completed successfully. Then, the maximum force value was lowered and the same simple manipulation was performed. In this case, the forces applied to the object were considered too high, so the micromanipulation process was automatically halted.

## 4. Discussion

Here, an intuitive and comprehensive platform for force-aided micromanipulation applications is presented. The several motion modes provide the user with a wide range of solutions that can be applied to multiple manipulation problems. The experiments performed are designed to showcase many of the system’s applications, but still keeping in mind possible future, more impactful applications.

The contact detection mode is a useful tool for precise manipulation and force application. As mentioned before, the part can be simply fixed in the workspace, allowing the μVBFS-P to apply a specific desired force. This is especially useful for mechanobiology studies, in which precise forces are applied to the membrane of a cell to study its development and responses to these forces. Additionally, this mode can be also used to calibrate the stiffness of new micro-force sensing probes. In this case, the uncalibrated sensor can remain stationary as a previously calibrated sensor applied known forces. As forces are applied and the deflections of the uncalibrated sensor measured, the system can be modeled as two springs in series (one from each sensor), thus allowing the stiffness calibration of the other sensor.

Moreover, the utilization of squeeze caging grasp transport primitive for micromanipulation applications greatly increases the success rate of the manipulation and diminishes the uncertainties during the process. As the object is lifted from the workspace, surface forces do not play a role in the manipulations, resulting in much more accurate motion when compared to standard push manipulation methods. Not only it allows for more accurate and robust manipulation, but it also incorporates a whole new dimension to the assembly process. As shown, the system is capable of 3D assembly, being able to stack micro-parts in the workspace.

As for the VR applications, it was shown that the system is able to reproduce a manipulation sequence input by a user via the VR environment. This is a great step towards more intuitive and automated micromanipulation tasks. In the future, multiple motion primitives can be recorded in a database through the VR environment with real tests using the physical system. The user will be able to select a motion primitive to apply to an object. For example, one can select to rotate the object by 90${}^{\circ }$ or move 200 μm to the right, and the system will have the proven instructions to perform said motion primitive in its database. Having such a system not only makes micromanipulation much easier and effective, but also provides solutions when it comes to training new users on the system (since the VR environment can simulate the physical system with more accuracy) and even remote micromanipulation, in which a user can look at the physical workspace through the VR environment and control it remotely from anywhere in the world.

In general, force-aided micromanipulation has many benefits when compared to standard micromanipulation. Firstly, the system can be used for delicate applications in which a maximum force has to be observed, as well as applications in the fields of mechanobiology or mechanical characterization. The ability to sense forces in real time provides the user with more control over the manipulation and a better feel for the process. This serves as a great platform for the development of autonomous manipulation systems, as they would be able to use the real-time force information to make decisions regarding the caging and manipulation, thus ensuring a safe, robust, and accurate system.

## Figures and Tables

**Table 1:** Properties of the μVBFSs.

Sensor	Direction	Stiffness (N/m)	Resolution (μN)	Range (μN)
I	x${}_{s}$	0.24	0.92	[0, 32]
	y${}_{s}$	0.43	1.65	[0, 65]
	z${}_{s}$	0.05	0.19	[0, 5]
II	x${}_{s}$	0.12	0.46	[0, 17]
	y${}_{s}$	0.24	0.93	[0, 37]
	z${}_{s}$	0.05	0.21	[0, 6]

Note: the resolution is based on the camera zoom (field of view) and its μm/pixel ratio.

**Table 2:** Accuracy tests comparison for caging versus pushing manipulation).

	Average Offset [μm]	Maximum Offset [μm]	Standard Deviation [μm]	Percent Error
Caging with Polygonal parts	15.46	23.45	1.83	7.73%
Caging with Circular parts	17.55	28.08	2.29	8.78%
Pushing Method	32.92	97.84	19.16	14.07%

## Supplementary Materials

The following are available online at https://www.mdpi.com/article/10.3390/mi12070784/s1, Figure S1: Sensor fabrication procedure schematic, Figure S2: VR system schematic.

## Abbreviations

The following abbreviations are used in this manuscript:

DOF	Degrees of Freedom
AFM	Atomic Force Microscope
μVBFS-MS	Vision-based Micro-Force Sensing Manipulation System
μVBFS-P	Vision-based Micro-Force Sensing Probe
VR	Virtual Reality
GUI	Graphical User Interface
CD	Contact Detection
PDMS	Polydimethylsiloxane
DRIE	Deep Reactive-Ion Etching
MEMS	Micro Electromechanical Systems
MOSSE	Minimum Output Sum of Squared Error
CSRT	Spatial Reliability Correlation Filter Tracker
ROI	Region of Interest
GPU	Graphics Processing Unit
