# Supplementary material for: Towards a Comprehensive and Robust Micromanipulation System with Force-Sensing and VR Capabilities
Source: Micromachines (Basel). 2021 Jun 30;12(7):784. doi: 10.3390/mi12070784 (PMC8307479; doi:10.3390/mi12070784)
Supplement: Supplementary file 1 [file micromachines-12-00784-s001.zip › micromachines-1253283-supplementary.pdf]

Towards a Comprehensive and Robust Micromanipulation System with Force-Sensing and VR Capabilities

Georges Adam, Subramaniam Chidambaram, Sai Swarup Reddy, Karthik Ramani, David J. Cappelleri

Supplemental Materials

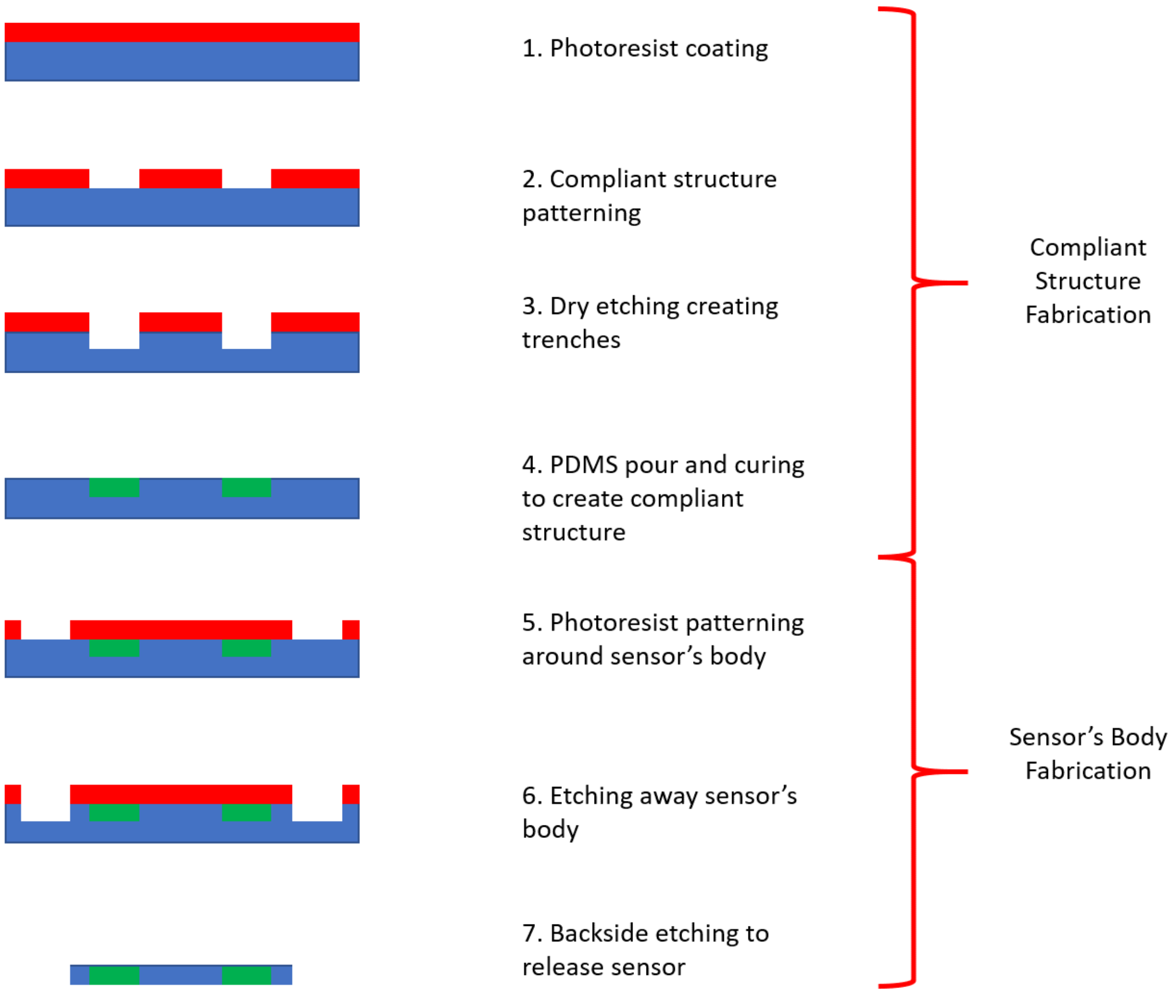

Figure S1. Schematic of the fabrication procedure of the μVBFS. The process is based on photolithography steps followed by deep reactive-ion etching (DRIE) to create trenches for the PDMS structure or to pattern the body of the sensor. Once both have been patterned, a backside etching is used to release it from the silicon wafer.

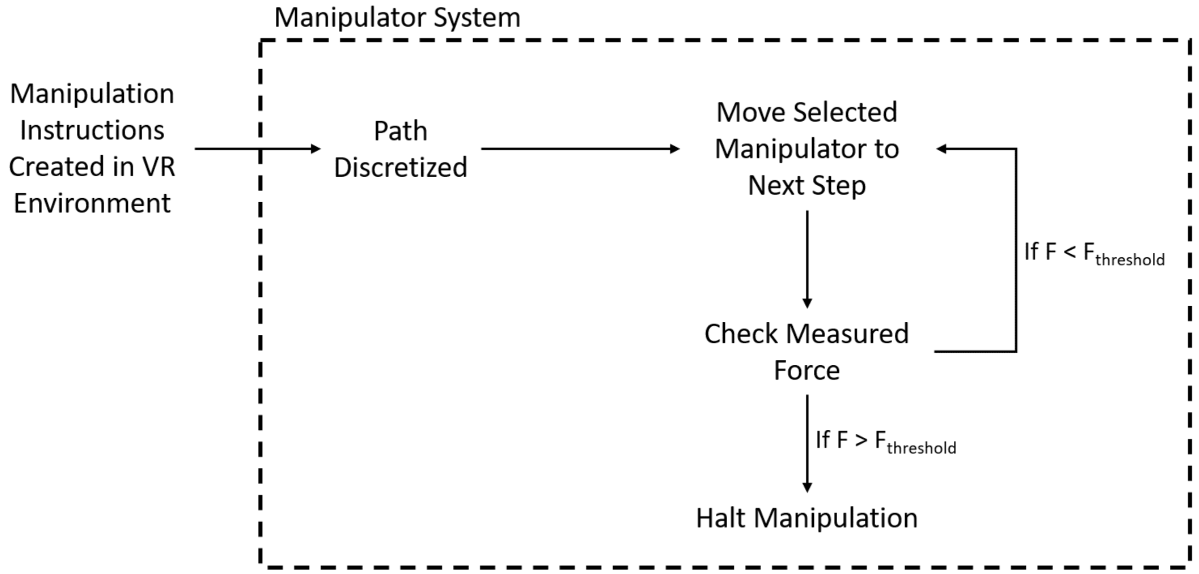

Figure S2. Schematic showing an overview of the VR experiments. After the path is discretized, the manipulator system keeps performing the desired instructions until manipulation is complete or the measured force is above the threshold force set by the user, thus ensuring safe manipulation.
